# 

**Published:** 1856-08

**Authors:** 


COMMUNICATIONS FOR PUBLICATION TO BE ADDRESSED TO 'EDITORIAL COMMITTEE.'

### IOWA
### MEDICAL JOURNAL.

CONDUCTED BY THE FACULTY OF THE

### COLLEGE OF PHYSICIANS AND SURGEONS

OF THE

### IOWA UNIVERSITY.

PUBLISHED EVERY ALTERNATE MONTH.

TERMS—TWO DOLLARS IN ADVANCE.

VOL. III. JULY AND AUGUST, 1856. NO. 4.

EDITORIAL COMMITTEE,

D. L. M'GUGIN, M. D.

JNO. R. ALLEN, M. D.

FINANCE COMMITTEE.

J. C. HUGHES, M. D.

KEOKUK, IOWA.

PRINTED AT THE POST BOOK AND JOB OFFICE.

BUSINESS COMMUNICATIONS TO THE 'FINANCE COMMITTEE.'

